# Bedside Flexible Cystoscopy-Guided First Suprapubic Catheter Exchange in a High-Risk Patient

**DOI:** 10.7759/cureus.116616

**Published:** 2026-09-20

**Authors:** Yoshihiro Kawaguchi, Shugo Koga

**Affiliations:** 1 Department of Urology, Morodomi Central Hospital, Saga, JPN; 2 Department of Cardiology, Morodomi Central Hospital, Saga, JPN

**Keywords:** bedside procedure, catheter exchange, flexible cystoscopy, guidewire, suprapubic catheter

## Abstract

Suprapubic catheter (SPC) exchange carries the risk of catheter malposition and tissue injury, particularly during the first exchange when the tract may not be fully matured. We report the case of a 56-year-old man with hypoxic-ischemic encephalopathy who was managed with mechanical ventilation and gastrostomy, had substantial suprapubic subcutaneous tissue (30 mm), and was receiving warfarin therapy. After initial 14-Fr SPC placement under ultrasonographic and flexible cystoscopic guidance, the first exchange on day 28 was performed at the bedside using flexible cystoscopy because transfer to the fluoroscopy suite was unfeasible. Direct visualization of the guidewire, catheter tip, and balloon confirmed intravesical positioning and allowed immediate monitoring for bleeding. The exchange was completed without significant bleeding or other complications. Bedside flexible cystoscopy may represent a practical and feasible option for the first SPC exchange in selected high-risk patients when fluoroscopic exchange is difficult, though further study is needed.

## Introduction

Suprapubic catheterization is a common urological procedure for long-term bladder drainage and is generally considered safe, offering an established alternative to long-term urethral catheters, particularly in patients experiencing urethral complications [1]. While initial suprapubic catheter (SPC) placement is performed surgically or percutaneously, the first catheter exchange - typically scheduled around four to six weeks post-placement - presents unique technical challenges. At this stage, the suprapubic tract may not be fully matured, which significantly increases the risk of false tract formation, catheter mispositioning, or unrecognized tissue injury during conventional blind exchange.

Although major complications during elective SPC management are overall uncommon [1], serious clinical problems can occur during catheter handling and exchange. Intestinal injury, particularly small bowel injury, represents an important type of SPC-related intestinal injury [2]. Moreover, catheter malposition during exchange can cause significant urethral injury when the balloon is inadvertently inflated outside the bladder [3]. These complications highlight the critical need to confirm catheter and balloon positioning during the first exchange.

Image-guided and cystoscopic techniques have been described to improve the safety of SPC placement in technically challenging situations [4]. However, reports specifically describing bedside flexible cystoscopy-guided first exchange of an SPC remain limited. Flexible cystoscopy offers a practical alternative at the bedside by providing direct visualization of the guidewire and balloon positioning while simultaneously allowing immediate assessment of intravesical and tract bleeding without requiring patient transport. This bedside approach is particularly valuable in "high-risk" patients - defined here as those with severe mobility limitations (e.g., mechanical ventilation dependency), altered anatomy (e.g., substantial suprapubic subcutaneous tissue), and active coagulopathy under systemic anticoagulation - for whom transfer to a fluoroscopy suite or conventional exchange carries substantial risk. Herein, we report a case of successful bedside flexible cystoscopy-guided first SPC exchange in such a high-risk patient for whom transfer to the fluoroscopy suite was unfeasible.

## Case presentation

A 56-year-old man with hypoxic-ischemic encephalopathy following aortic dissection had been hospitalized for approximately 1.5 years and was managed with a mechanical ventilator, gastrostomy, and long-term urethral catheterization (Table 1).

**Table 1:** Patient background, laboratory findings, and clinical course of the first suprapubic catheter exchange under flexible cystoscopic guidance. Relevant patient characteristics, laboratory findings, and procedural details are summarized. SPC, suprapubic catheter; PT-INR, prothrombin time-international normalized ratio

Time point	Clinical status/Procedure	Findings	Reference range
Patient background	Body weight	81.1 kg	-
	Subcutaneous tissue thickness at the suprapubic region	Approximately 30 mm	-
	Anticoagulation	Warfarin use	-
	Mechanical ventilation	Yes	-
	Gastrostomy	Yes	-
Before SPC placement	Urinary management	Urethral catheterization	-
SPC placement	Initial suprapubic catheter placement	14-Fr SPC	-
Before first exchange	Clinical status	Ventilator-dependent; warfarin use	-
	White blood cell count	4,500/μL	3500-9800
	Hemoglobin	10.2 g/dL	13.5-17.6
	Platelet count	203,000/μL	130,000-369,000
	C-reactive protein	2.0 mg/dL	0-0.30
	PT-INR	2.46	0.85-1.15
Day 28 after SPC placement	First SPC exchange	Bedside flexible cystoscopy-guided exchange; approximately 10 min; successful without complications	-
Immediately after exchange	Cystoscopic confirmation	Balloon appropriately positioned within the bladder	-
Next day	Follow-up	Urine drainage maintained; no significant hematuria or other complications	-

He was receiving warfarin therapy for a mechanical heart valve. The patient presented with purulent urethral discharge, a tendency toward hypospadias, and marked systemic inflammation with a C-reactive protein (CRP) level of 20.77 mg/dL. Urine culture identified *Klebsiella pneumoniae *and *Pseudomonas aeruginosa*, whereas computed tomography revealed no other apparent infectious focus (Figure 1). Targeted intravenous antibiotic therapy with meropenem (0.5 g twice daily) was initiated, resulting in marked clinical improvement and resolution of urethritis. Therefore, SPC was selected for definitive urinary diversion.

**Figure 1:**
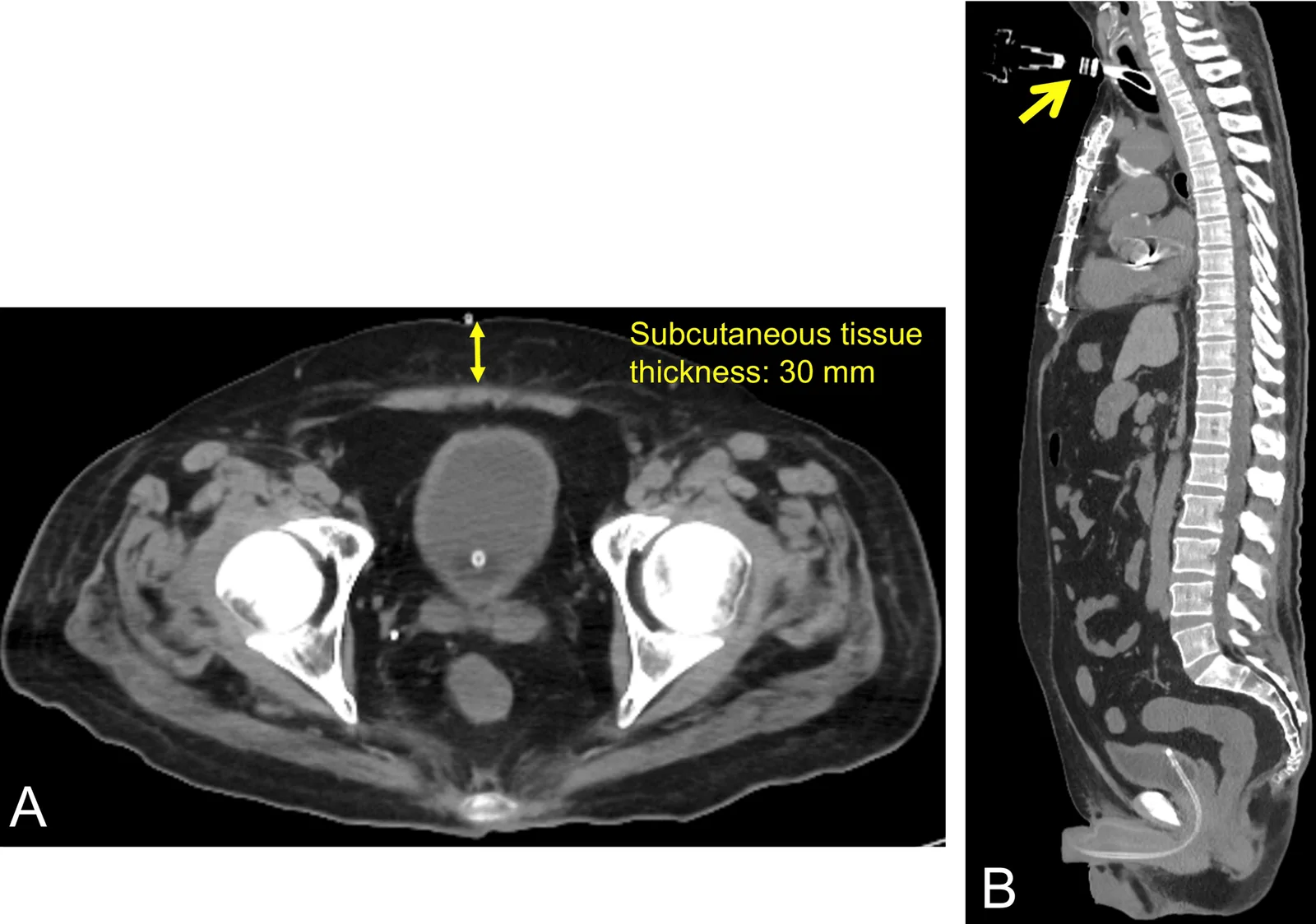
Pre-procedural computed tomography findings. (A) Axial computed tomography image showing approximately 30 mm of subcutaneous tissue between the skin surface and the anterior rectus abdominis fascia in the suprapubic region (yellow double-headed arrow). (B) Sagittal computed tomography image showing an indwelling urethral catheter with the balloon positioned within the urinary bladder and an endotracheal tube (yellow arrow).

Initial 14-Fr SPC placement was performed under general anesthesia with ultrasonographic and flexible cystoscopic guidance after temporary discontinuation of warfarin with intravenous heparin bridging. Following clinical stabilization, the first SPC exchange was scheduled on day 28 post-placement, a time point when the suprapubic tract may not be fully matured. On the day of the first exchange, the inflammation had resolved, with a CRP level of 2.0 mg/dL and a white blood cell count of 4,500/μL.

Because of mechanical ventilation, gastrostomy, active coagulopathy, and substantial suprapubic subcutaneous tissue (measured as 30 mm from the skin surface to the anterior rectus abdominis fascia), transfer to the fluoroscopy suite and conventional blind catheter exchange were considered unsafe and unfeasible. To avoid the thromboembolic risks associated with a mechanical heart valve, warfarin was continued without interruption for the first exchange, resulting in an elevated prothrombin time-international normalized ratio (PT-INR) of 2.46.

Laboratory findings on the day of the first SPC exchange are summarized in Table 1. Under aseptic conditions with the patient in the supine position, topical anesthesia with approximately 5 g of 2% lidocaine gel was applied into the tract and urethra, and a 14-Fr balloon catheter exchange was performed at the bedside under flexible cystoscopic guidance without additional prophylactic systemic antibiotics. A 0.038-inch × 150 cm Sensor Straight Tip Guidewire (Boston Scientific, Marlborough, MA) was inserted through the existing suprapubic catheter under direct flexible cystoscopic visualization (Figure 2).

**Figure 2:**
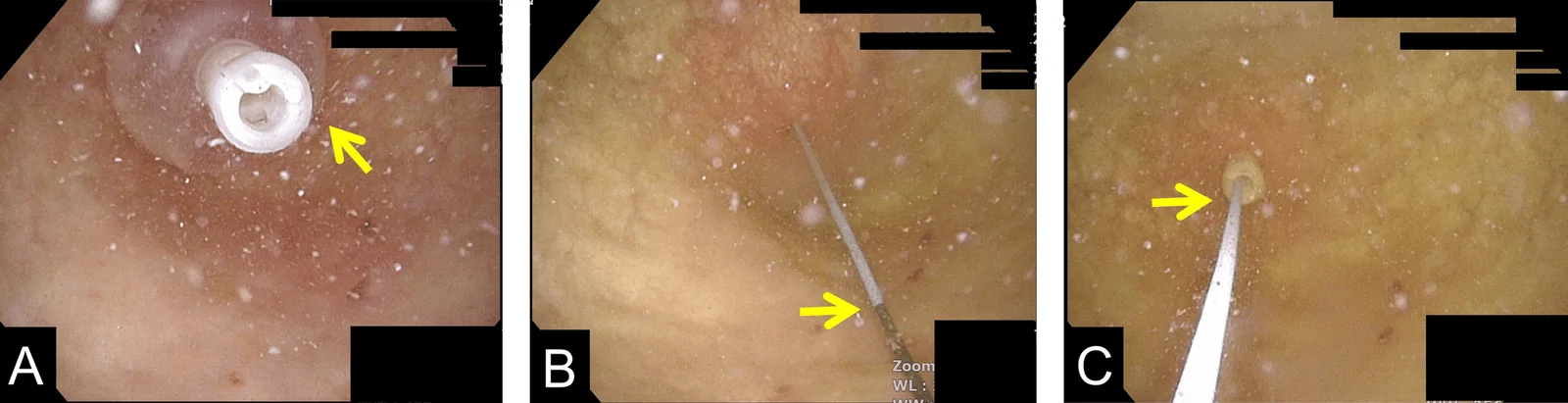
Cystoscopic findings during bedside suprapubic catheter exchange. (A) The balloon of the existing suprapubic catheter was inflated within the urinary bladder (yellow arrow). (B) A guidewire (Sensor Straight Tip Guidewire; Boston Scientific, Marlborough, MA) was advanced through the existing suprapubic catheter, with the distal tip of the guidewire directly visualized within the bladder (yellow arrow). (C) A new 14-Fr suprapubic catheter was advanced over the guidewire into the bladder while the bladder mucosa was monitored for bleeding under direct flexible cystoscopic visualization (yellow arrow).

The existing SPC was removed, and the sensor guidewire was advanced through the suprapubic tract under continuous cystoscopic visualization. The guidewire, including its relatively rigid distal portion, was directly monitored within the bladder, followed by advancing a new 14-Fr catheter over the guidewire. After cystoscopic confirmation of the catheter tip and balloon within the bladder lumen, the balloon was inflated with 7 mL of sterile water. Direct cystoscopy confirmed the absence of significant intravesical or suprapubic tract bleeding (Figure 3). The exchange procedure was completed in approximately 10 minutes.

**Figure 3:**
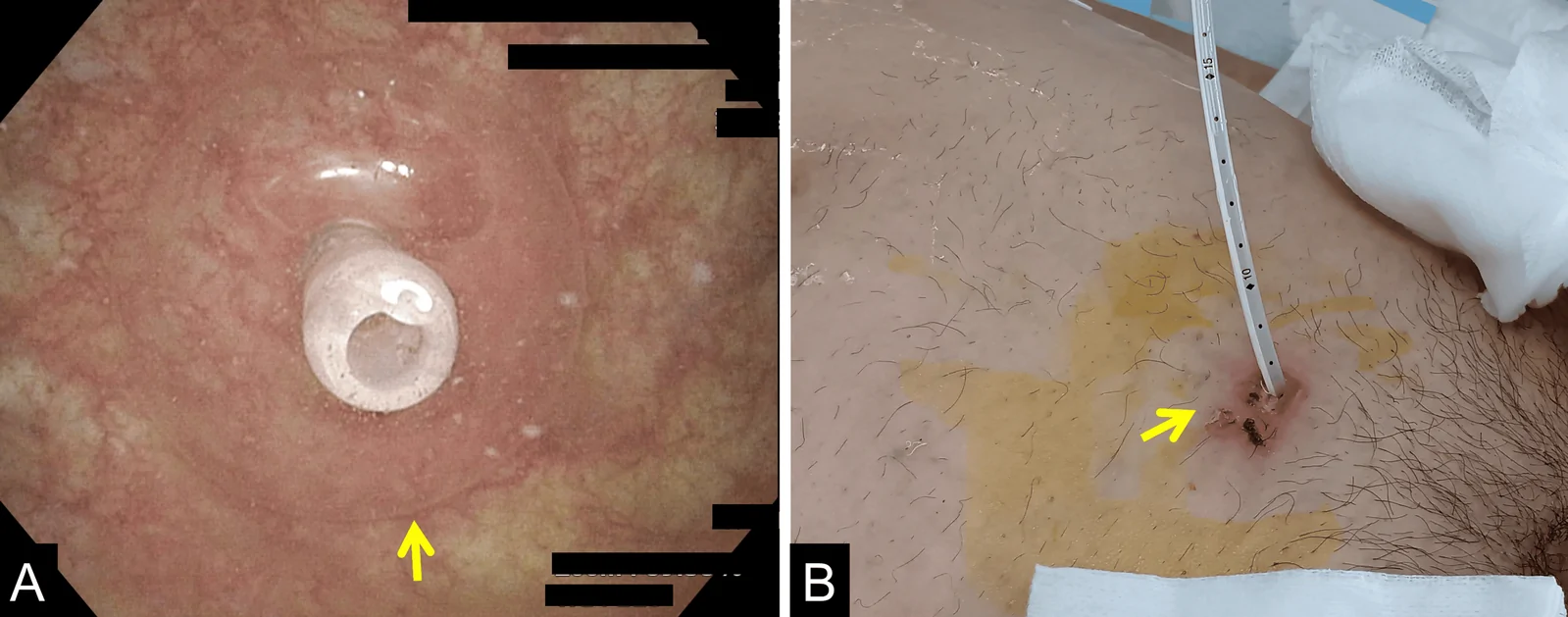
Findings after suprapubic catheter exchange. (A) The balloon cuff of the newly placed 14-Fr suprapubic catheter was inflated within the urinary bladder, with no apparent bleeding observed on cystoscopic examination (yellow arrow). (B) The skin site after catheter exchange showed no apparent bleeding or other local complications (yellow arrow).

On the following day, the patient maintained adequate urine drainage without macroscopic hematuria, hemoglobin drop, or procedural complications. Over an extended follow-up period of several months, the patient demonstrated stable vital signs, clear urine output, and no evidence of delayed tract disruption, local wound infection, or visceral injury.

## Discussion

Our case experience provides two important clinical lessons. First, in patients for whom transfer to a fluoroscopy suite or conventional catheter exchange is difficult because of mechanical ventilation, gastrostomy, or substantial suprapubic soft tissue, bedside flexible cystoscopy may facilitate safe first SPC exchange by allowing direct confirmation of catheter position. Second, in patients with an elevated INR receiving warfarin, flexible cystoscopy allows direct assessment of intravesical and suprapubic tract bleeding and visualization of the guidewire during advancement, which may help reduce the risk of unrecognized tissue injury.

SPC is generally considered safe for long-term bladder drainage; however, serious complications can occur. In a series of 1,000 elective SPC insertions, major complications were uncommon [1], but a recent scoping review identified intestinal injury, particularly small bowel injury, as an important SPC-related complication [2]. Importantly, bowel injury may occur or become apparent during catheter exchange. Small bowel injury associated with the first SPC exchange has been documented [5,6], and urethral injury caused by inadvertent balloon inflation outside the bladder during exchange has been reported [3]. These reports highlight the importance of confirming catheter and balloon position during the first exchange.

Image-guided and cystoscopic techniques have previously been described to improve the safety of SPC placement. A technique combining ultrasonography and flexible cystoscopy facilitates safer SPC insertion [7], while laparoscopic- and cystoscopy-assisted insertion is used in patients whose anatomy is challenging [4]. Severe bowel injury following repeated unguided SPC catheterization has been reported in a patient with previous pelvic radiotherapy, emphasizing the potential value of image-guided procedures in high-risk patients [8].

In our patient, bedside flexible cystoscopy allowed direct confirmation of the catheter tip and balloon within the bladder without transfer to the fluoroscopy suite. This was particularly useful because the patient was mechanically ventilated, had a gastrostomy, and had substantial suprapubic soft tissue. Furthermore, despite warfarin therapy and an elevated INR, direct cystoscopic visualization enabled monitoring for intravesical bleeding and confirmation of guidewire position during the procedure. Although this single case cannot confirm that cystoscopic guidance prevents bleeding or tissue injury, our findings suggest that bedside flexible cystoscopy may be a practical safety measure for selected high-risk patients undergoing first SPC exchange. As this is a single case report, further experience is needed to establish the safety and utility of this approach.

## Conclusions

Bedside flexible cystoscopy-guided first SPC exchange may be useful for selected high-risk patients when transfer to a fluoroscopy suite is difficult. Direct visualization of the catheter and balloon may facilitate confirmation of intravesical positioning. It may also allow early recognition of bleeding or other procedural complications during catheter exchange.

## References

[1] Hobbs C, Howles S, Derry F, Reynard J (2022). Suprapubic catheterisation: a study of 1000 elective procedures. BJU Int.

[2] Jarral F, Hamdy A, Abusand O, Mobayen R, Tokidis E (2024). Intestinal injury after suprapubic catheterisation: a scoping review. Cureus.

[3] Hatoum ZG, Alter SM (2020). A Foley folly: unintended proximal urethral catheterization and balloon inflation causing urethral injury during suprapubic cystostomy exchange. J Am Coll Emerg Physicians Open.

[4] Tourky M, Knight R, Rajagopal R (2015). Laparoscopic and cystoscopic-assisted suprapubic catheter insertion: a case study and technical note. Arab J Urol.

[5] Barai KP, Islam S (2009). Suprapubic catheterization complicated by an iatrogenic enterocutaneous fistula: a case report. Cases J.

[6] Jackson BL, Leeder PC, Williams JH (2010). Delayed presentation of small bowel injury during suprapubic catheterisation. J Surg Case Rep.

[7] Lawrentschuk N, Lee D, Marriott P, Russell JM (2003). Suprapubic stab cystostomy: a safer technique. Urology.

[8] Verma S, Vaka SK, Jain A, Reddy A, Nelamangala Ramakrishnaiah VP (2020). Inadvertent bowel injury following repeated suprapubic catheterisation in a patient of post-pelvic radiotherapy. Cureus.

